# Oral Ingestion of Deep Ocean Minerals Increases High-Intensity Intermittent Running Capacity in Soccer Players after Short-Term Post-Exercise Recovery: A Double-Blind, Placebo-Controlled Crossover Trial

**DOI:** 10.3390/md17050309

**Published:** 2019-05-24

**Authors:** Matthew F. Higgins, Benjamin Rudkin, Chia-Hua Kuo

**Affiliations:** 1 Human Sciences Research Centre, University of Derby, Kedleston Road, Derby DE22 1GB, UK; benrudkin_345@hotmail.com; 2 Institute of Sports Sciences, University of Taipei, Shilin District, Taipei 111, Taiwan; kuochiahua@gmail.com

**Keywords:** deep ocean minerals, soccer, football, high-intensity intermittent running, exercise capacity, recovery

## Abstract

This study examined whether deep ocean mineral (DOM) supplementation improved high-intensity intermittent running capacity after short-term recovery from an initial bout of prolonged high-intensity running in thermoneutral environmental conditions. Nine healthy recreational male soccer players (age: 22 ± 1 y; stature: 181 ± 5 cm; and body mass 80 ± 11 kg) completed a graded incremental test to ascertain peak oxygen uptake (V^·^O_2PEAK_), two familiarisation trials, and two experimental trials following a double-blind, repeated measures, crossover and counterbalanced design. All trials were separated by seven days and at ambient room temperature (i.e., 20 °C). During the 2 h recovery period after the initial ~60 min running at 75% V^·^O_2PEAK_, participants were provided with 1.38 ± 0.51 L of either deep ocean mineral water (DOM) or a taste-matched placebo (PLA), both mixed with 6% sucrose. DOM increased high-intensity running capacity by ~25% compared to PLA. There were no differences between DOM and PLA for blood lactate concentration, blood glucose concentration, or urine osmolality. The minerals and trace elements within DOM, either individually or synergistically, appear to have augmented high-intensity running capacity in healthy, recreationally active male soccer players after short-term recovery from an initial bout of prolonged, high-intensity running in thermoneutral environmental conditions.

## 1. Introduction

Hydrothermal systems in the deep ocean have been proposed to be among the most credible settings for the biological origins of life [1,2]. Indeed, such alkaline systems have been suggested as the source of the evolution of primordial metabolism [1,3]. The marine environment possesses a rich pool of bioactive ingredients with numerous purported health benefits [4]. Moreover, novel marine-based bioactive compounds might also augment human performance, especially in those who engage in physical activity [4]. Administration of deep ocean minerals (DOM) has previously demonstrated positive effects on exercise performance in animal [5,6] and human models [7,8,9]. For example, Hou et al. [7] reported that DOM extracted from 662 m below the ocean surface facilitated substantially quicker recovery from a prolonged bout of exercise at 30 °C, which induced 3% reduction in body mass of healthy males. More specifically, maximal oxygen uptake (V^·^O_2MAX_) increased by ~2% after 4 h compared to baseline with DOM, whilst it was reduced by ~11% with placebo. After 24 h, V^·^O_2MAX_ increased by ~6% compared to baseline with DOM, whilst it was ~13% lower with placebo. The authors opined that specific elements in DOM, such as boron, magnesium and rubidium, were likely to have contributed to the observed ergogenic effects. Interestingly, using a similar protocol, Stasiule et al. [9] reported comparable differences in aerobic capacity of healthy females 4 h after supplementation with (deep) mineral water (DMW) extracted from a well at a depth of 689 m or placebo (purified tap water). Similarly, these authors also postulated that the minerals and trace elements that constituted DMW may have worked collaboratively to recover normal human performance [9].

Given the importance of optimising recovery for individuals who train/compete multiple times per day with limited time between bouts of exercise [10,11], and the fact that it is presently unknown whether DOM can improve high-intensity intermittent running capacity, which is integral to many team sports such as soccer [12], this study examined the effects of DOM on high-intensity intermittent running capacity in soccer players after short-term recovery from an initial bout of prolonged high-intensity exercise in thermoneutral environmental conditions. As such, the primary performance outcome measure for this study was high-intensity intermittent running endurance capacity with secondary outcomes including various metabolic (i.e., blood glucose and blood lactate), anthropometric (i.e., body mass), and physiological (i.e., urine osmolality) markers. We hypothesised that DOM would augment high-intensity intermittent running endurance capacity with other variables remaining similar between treatments.

## 2. Results

### 2.1. Graded Incremental Test

At the end of the graded increment test, mean relative V^·^O_2_, heart rate (HR), ratings of perceived exertion (RPE), and respiratory exchange ratio (RER) were 46 ± 4 mL kg min^−1^, 197 ± 4 beats min^−1^, 19.3 ± 0.7, and 1.07 ± 0.05, respectively.

### 2.2. Initial Fatiguing Protocol

There were no differences between the DOM and PLA trials for the time covered during the initial running bout at 75% V^·^O_2PEAK_ (58 ± 4 min vs. 58 ± 4 min, respectively; P = 1.00, r = 0) or the subsequent intermittent running until volitional exhaustion (163 ± 53 s vs. 160 ± 52 s, respectively; P = 0.92, d = 0.05; −0.87, 0.98). There was no interaction (P = 0.45, Pη^2^ = 0.1) or main effect of treatment (P = 0.31, Pη^2^ = 0.1) for HR during the bout of running at 75% V^·^O_2PEAK_. However, there was a main effect for time (P < 0.001, Pη^2^ = 0.8; Table 1). There was no interaction (P = 0.23, Pη^2^ = 0.2) or main effect of treatment (P = 0.09, Pη^2^ = 0.3) for RPE_L_ during the bout of running at 75% V^·^O_2PEAK_, although there was a main effect for time (P < 0.001, Pη^2^ = 0.9; Table 1). There was no interaction (P = 0.09, Pη^2^ = 0.2) or main effect of treatment (P = 0.09, Pη^2^ = 0.3) for RPE_O_ during the running at 75% V^·^O_2PEAK_; however, there was a main effect for time (P < 0.001, Pη^2^ = 0.9; Table 1).

### 2.3. Post-Recovery Protocol

All participants completed 20 min post-recovery running at 75% V^·^O_2PEAK_ for both experimental trials. There were no interactions (P = 0.60, Pη^2^ = 0.1; P = 0.95, Pη^2^ = 0.01; P = 0.94, Pη^2^ = 0.02) or main effects of treatment (P = 0.61, Pη^2^ = 0.03; P = 0.69, Pη^2^ = 0.02; P = 0.49, Pη^2^ = 0.1) for HR, RPE_L_ and RPE_O_, respectively, during the 20 min running at 75% V^·^O_2PEAK_ post-recovery; however, there were main effects of time for all variables (all P < 0.001, Pη^2^ = 0.8; Table 2).

### 2.4. High-Intensity Intermittent Running Capacity Test

**Figure 1:**
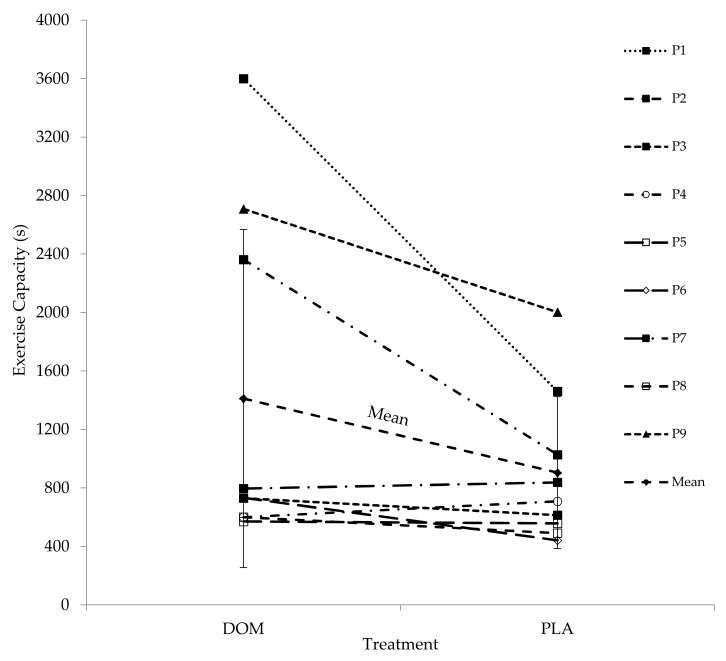
Individual and group mean high-intensity intermittent running capacity after consuming deep ocean minerals (DOM) or placebo (PLA).

There was no clear order effect between Trials 1 and 2 of the high-intensity intermittent running capacity test (1048 ± 713 s vs. 1268 ± 1103 s; P = 0.21, r = 0.4). In contrast, DOM increased exercise capacity by 56% (1411 ± 1157 s vs. 905 ± 520 s; P = 0.038, r = 0.7) compared to PLA (Figure 1). 

Due to the variability in exercise capacity between treatments and participants, HR and perceptual data were only analysed for the first four shuttles at 90% V^·^O_2PEAK_ and at volitional exhaustion. There was no interaction (P = 0.09, Pη^2^ = 0.2) or main effect of treatment (P = 0.68, Pη^2^ = 0.02) for HR; however, there was a main effect for time (P < 0.001, Pη^2^ = 0.5) with HR greater at the end of exercise (174 ± 7 beats min^−1^) compared to after 1 min (168 ± 7 beats min^−1^, P < 0.02) and 2 min (169 ± 6 beats min^−1^, P < 0.05). Furthermore, there were no interactions (P = 0.86, Pη^2^ = 0.02; P = 0.09, Pη^2^ = 0.2) or main effects of treatment (P = 0.86, Pη^2^ < 0.01; P = 0.4, Pη^2^ = 0.09) for RPE_L_ and RPE_O_, respectively. As with HR, there were main effects of time for RPE_L_ (P = 0.001, Pη^2^ = 0.6) and RPE_O_ (P < 0.001, Pη^2^ = 0.7), whereby RPE_L_ (9.4 ± 0.7) and RPE_O_ (19.2 ± 1.2) were greater at volitional exhaustion compared to after 1 min (6.9 ± 1.6, 15.7 ± 2.0; P < 0.01) and 2 min (7.4 ± 1.6, 16.4 ± 2.1; P < 0.05), respectively.

### 2.5. Urine Osmolality

There was no interaction (P = 0.18, Pη^2^ = 0.2) or main effect of treatment (P = 0.88, Pη^2^ < 0.01) or time (P = 0.12, Pη^2^ = 0.3) for urine osmolality (Table 3).

### 2.6. Body Mass

There was an interaction for body mass (P < 0.02, Pη^2^ = 0.4), whereby body mass was greater for DOM vs. PLA (80.1 ± 11.3 vs. 79.8 ± 11.2 kg, respectively; P < 0.05, d = 0.03; −0.90, 0.95) at baseline only (Table 4). There was also a main effect for time (P < 0.001, Pη^2^ = 0.8), whereby body mass was greater (all P < 0.001) at baseline (80.0 ± 10.9 kg) compared to after 60 min running at 75% V^·^O_2PEAK_ (79.3 ± 10.9 kg) after 2 h recovery (79.4 ± 11.0 kg) and at the end of the exercise capacity test (79.3 ± 10.9 kg). There was no main effect for treatment (P = 0.84, Pη^2^ < 0.01).

### 2.7. Blood Lactate

There was no interaction (P = 0.65, Pη^2^ = 0.07) or main effect of treatment (P = 0.74, Pη^2^ = 0.01) for blood lactate; however, there was a main effect for time (P = 0.001, Pη^2^ = 0.6; Table 5).

### 2.8. Blood Glucose

There was no interaction (P = 0.43, Pη^2^ < 0.1) or main effect of treatment (P = 0.24, Pη^2^ = 0.2) or time (P = 0.13, Pη^2^ = 0.2) for blood glucose (Table 5).

## 3. Discussion

Oral ingestion of DOM has previously demonstrated positive effects on exercise performance in humans [7,8,9], although such benefits have been observed during exercise after recovery from an initial bout of prolonged dehydrating exercise in the heat (i.e., 30 °C). Therefore, the aim of this study was to examine whether DOM facilitated ergogenic benefits during exercise after recovery from an initial bout of prolonged exercise in more thermoneutral conditions (i.e., 20 °C). In support of our hypothesis, DOM increased high-intensity intermittent running capacity in recreationally active soccer players at the group level by 56%. However, assuming a conservative daily variation of ~10% for a high-intensity exercise capacity test [13], and after accounting for any potential order effects between Trials 1 and 2 of ~20%, we suggest the minimum likely ergogenic effects after DOM at the group level to be ~25%. In summary, oral ingestion of DOM seems likely to promote ergogenic benefits for recreationally active soccer players after recovery from prolonged exercise in thermoneutral environmental conditions; however, given the somewhat variable results, an individual approach to supplementation is warranted.

In the present study, body mass was greater at baseline compared to after 60 min running at 75% V^·^O_2PEAK_ after 2 h recovery and at the end of the high-intensity intermittent exercise capacity test. The majority of this reduction in body mass over time is most likely indicative of sweat losses. As prolonged endurance exercise might induce an increase in magnesium (Mg) excretion via sweat and urine [6], it is plausible that participants in the present study exhibited marked Mg losses. Additionally, amongst several other factors such as decreases in food crop magnesium content and the availability of refined and processed foods, the vast majority of people in modern societies are at risk for magnesium deficiency regardless. Furthermore, a normal serum Mg level does not necessarily rule out Mg deficiency [14]. As Mg is involved with numerous fundamental biological processes, such as energy production, electrolyte regulation and oxygen uptake, and with evidence that marginal Mg deficiency impairs performance and augments the negative consequences of strenuous exercise [15], it seems likely that the high Mg concentration in DOM (Table 6) has played some role in the observed ergogenic effects. Indeed, the mean amount of Mg consumed from DOM in the present study (~236 mg) was ~80% of the participant’s 300 mg recommended daily allowance (RDA) [16]. Moreover, a recent review provides further support for the potential of magnesium supplementation to improve aspects related to both aerobic and anaerobic exercise [17].

Boron (B) has been shown to boost Mg absorption, reduce levels of inflammatory biomarkers such as high-sensitivity C-reactive protein (hs-CRP), tumour necrosis factor α (TNF-α), and interleukin 6 (IL-6), as well as contribute to the synthesis and activity of crucial biomolecules such as nicotinamide adenine dinucleotide (NAD^+^; [18]). Therefore, the B in DOM could work synergistically with elevated levels of Mg, or it could work individually through the mediation of exercise-induced inflammation and/or through facilitating key biochemical reactions where NAD^+^ is crucial, such as ATP production and calcium signalling [18]. Albeit in an older population and using a different exercise modality than the present study, we have previously shown that DOM can mediate the post-exercise neutrophil to lymphocyte ratio (NLR; [19]), which is associated with various cytokines including IL-6 [20]. Furthermore, both acute (one day) and chronic (seven days) B supplementation has been shown to be tolerated well in humans with chronic supplementation displaying large reductions in inflammatory cytokines and a large increase in free testosterone [21]. However, it should be added that the mean dosage used in the present study (~0.74 mg) was substantially less than the acute dosage used in the aforementioned study (10 mg; [21]), and that using similar hardness of DOM (713) to that of the present study (704), Hou et al. [7] reported no differences in testosterone between DOM and PLA.

Rubidium (Rb), present in all human tissues and ranging from 8 to 30 mg·kg^−1^ [22], has been referred to as a ‘biological proxy’ for potassium (K^+^) [23]. It is well known that there is a rapid and marked increase of extracellular K^+^ and considerable loss of K^+^ from active muscle during high-intensity exercise. Thus, it is likely that the resultant electrical changes induced by ionic shifts across the muscle surface membrane are principal contributors of fatigue [24]. Therefore, it is possible that the high levels of Rb in DOM (Table 6) might proxy for K^+^ and facilitate a prolonged status of optimal electrical activity and, hence, contraction in the muscle, thus delaying fatigue. Furthermore, Rb has been found to be 49% and 34% greater in professional footballers than sedentary controls and long-distance runners, respectively [25]. However, although Rb is associated with the K cycle, it does not necessarily substitute for it [22,25] and, thus, other mechanisms might be responsible. Besides, Rb has been implicated in brain function, although specific roles are yet to be identified [22,25].

In support of our hypothesis, the present study found no differences in urine osmolality between treatments. This is in contrast to Keen et al. [8] who reported that DOM returned salivary osmolality to baseline values approximately two-fold quicker than spring water and a carbohydrate-based sports drink. However, a variety of methodological differences between studies are likely to explain the observed difference. For example, the participants in the Keen et al. [8] study had exercised in the heat to achieve approximately −3% reduction in body mass, and subsequent rehydration was based on prescriptive rather than ad libitum intake. Furthermore, salivary osmolality demonstrates considerable day-to-day measurement variability and in magnitude of response to hypohydration; thus, its use as a method of assessing hydration status has been questioned [26]. However, despite differences in urine osmolality between studies, Keen et al. [8] also reported physical performance improvement with DOM. Ingesting DOM facilitated the greatest recovery of post-exercise knee extension peak torque, although participants failed to fully recover baseline peak torque regardless of treatment. In contrast to our original hypothesis, we observed a difference in body mass at baseline between DOM and PLA. However, the difference of 0.28 kg was well below the typical daily variation of body mass measurement in young males (0.6 kg) previously reported [27]. Thus, we do not believe differences in body mass had any major influence on our results.

In support of our hypothesis, there were no differences in blood lactate and blood glucose between DOM and PLA. This was similar to Wei et al. [19], who reported no differences in either metabolite after 15 min cycling at 75% V^·^O_2MAX_. Importantly the absolute values of blood lactate after 60 min running at 75% V^·^O_2PEAK_ in the present study were similar to those observed in various other studies examining blood lactate responses in the second half of a soccer match (3.7 to 4.7 mmol L^−1^; [28]), highlighting that our protocol had a similar metabolic profile. However, with various methodological differences between studies and a lack of other data, the influence of DOM on blood lactate and blood glucose requires further investigation. 

Arguably the most intriguing finding from the metabolic data relates to a specific participant who, after completion of this study, was diagnosed with type I diabetes. The participant confirmed there was a familial history of this, which was undisclosed prior to participation (P2; Table 5, bottom panel). Strikingly, at the end of the recovery period, blood glucose had increased by 65% (+3.69 mmol L^−1^) after DOM but 117% for PLA (+8.23 mmol L^−1^) when compared to values at the end of 60 min running at 75% V^·^O_2PEAK_. Excluding P2, the mean increase in blood glucose between these time points was 18% and 22% for DOM and PLA, respectively. Furthermore, only part of the difference observed for P2 might be explained by the differences between treatments of −1.39 mmol L^−1^ at baseline and of +1.35 mmol L^−1^ after completion of 60 min running at 75% V^·^O_2PEAK_ for PLA vs. DOM, respectively. The potential of DOM to facilitate greater glucose uptake is supported by Ha et al. [29], who reported that DOM improved glucose tolerance and suppressed hyperglycaemia via modulation of glucose metabolism in streptozotocin-induced diabetic mice. The potential antidiabetic effects of DOM (or derivatives of) have also been reported in various other animal models [30,31,32]. Furthermore, a number of the elements contained in DOM, such as vanadium [33] and chromium [34], have also been strongly linked to improved glucose metabolism. 

This research has a number of strengths. Firstly, we are the first to report ergogenic benefits after oral ingestion of DOM in humans in thermoneutral environmental conditions; thus, we expand on the potential pool of individuals who might benefit from this approach. Additionally, by incorporating fluid consumption based on an individual participant’s preference in familiarisation trials, we feel this provides wider applied ecological validity. This research is also not without limitation. Even though the sample size is consistent with seminal papers in this field [7,9], we acknowledge the relative lack of statistical power. Moreover, we acknowledge that the analysis on the type I diabetic participant is somewhat speculative and, thus, should be treated with caution. That said, we believe it could serve as useful initial case study data and therefore could be a translational platform to research examining the potential effects of DOM on (dysfunctional) glucose metabolism in humans during/post exercise.

In summary, in support of our hypothesis, DOM increased high-intensity intermittent running capacity in soccer players after short-term recovery from an initial bout of prolonged exercise at the group level by ~25%. To date, the mechanism for this effect is unclear, and further work is required to establish the mechanism(s) behind the ergogenic effects of DOM to maximise its potential performance and health benefits in the wider community.

## 4. Materials and Methods 

### 4.1. Participants

After reading an information sheet about the proposed research, and subsequently providing written informed consent, nine healthy, nonelite male soccer players (age: 22 ± 1 y; stature: 181 ± 5 cm; body mass 80 ± 11 kg; mean weekly soccer-specific training time: 134 ± 64 min (range: 40 to 240); and mean nonsoccer-specific training time (e.g., gym/outdoor running): 37 ± 44 min (range: 0 to 90)) volunteered to participate in the study, which had received university ethical approval (reference: S2BR2016). The experimental cohort was recruited via opportunistic and convenience sampling. Participants were eligible if they were nonelite soccer-playing males who were healthy, free from injury, and were between 18 and 30 years of age. 

### 4.2. Experimental Design

Participants completed five visits to the laboratory. Firstly, they completed a graded incremental test to ascertain peak oxygen uptake (V^·^O_2PEAK_) followed by two familiarisation trials. Using a repeated measures, crossover, and counterbalanced design, participants then completed two experimental trials. All trials were completed on a motorised treadmill (Mercury S, Woodway, Waukesha, WI, USA), separated by 7 d, undertaken at ambient room temperature (i.e., ~20 °C), and commenced at similar times of day (e.g., 9 am and 10 am) to minimise any potential circadian rhythmic effects on soccer-specific endurance [35].

### 4.3. Pre-Experimental Procedures

Participants refrained from strenuous physical activity and alcohol for at least 24 h prior to exercise. Participants were also requested to keep a written record of their dietary intake 24 h before their first trial and to replicate this before all remaining trials. Participants were also asked to avoid consuming any dietary supplements that might influence exercise (e.g., caffeine) 24 h prior to each trial. Due to the extended washout period, participants were verbally screened for the use of beta-alanine and creatine monohydrate supplementation. No participant was excluded from the study due to prior ingestion of either.

On the first visit to the laboratory, the participant’s age, stature (cm), and body mass (kg) were recorded, with only body mass being recorded on subsequent visits. One hour prior to exercise, participants were asked to ingest 500 mL of tap water to ensure an adequate and consistent level of hydration status [36]. Hydration status was measured via urine osmolality (Pocket Refractometer, Atago, Tokyo, Japan) and adequate hydration was deemed as <600 mOsmol·Kg^−1^ [37]. If participants did not achieve this, they were required to slowly consume water during 15 min seated rest before being re-tested. If urine osmolality was found to be still >600 mOsmol·Kg^−1^, participants were excluded from the trial. Two participants required small amounts of water in addition to the 500 mL bolus, but all participants were found to be adequately hydrated before each trial. A heart rate (HR) monitor (FS3c, Polar, Finland) was subsequently attached. After 5 min seated rest, HR was recorded, and a finger prick capillary blood sample was taken and subsequently analysed for blood lactate (BLa) and blood glucose (BGl) concentrations (Biosen C_line, EKF Diagnostic, Magdeburg, Germany).

### 4.4. Graded Incremental Test

Assessment of V^·^O_2PEAK_ was achieved using an incremental exercise test on a motorised treadmill (Mercury S, Woodway, Waukesha, WI, USA). After completing an initial 5 min warm up at 8 km h^−1^, participants commenced running at 9 km h^−1^ for 3 min. Incremental increases of 1 km h^−1^ were applied every three minutes until volitional exhaustion. At the end of each stage, and upon completion of exercise, HR was telemetrically recorded, and ratings of perceived exertion (6–20; [38]) were collected. Expired breath-by-breath gas (Cortex, Metalyser II, Leipzig, Germany) was recorded throughout the test and subsequently analysed for oxygen consumption (V^·^O_2_) and the respiratory exchange ratio (RER) over the last 60 s of rest and the last 30 s of exercise.

### 4.5. Familiarisation Trials

After collection of initial baseline measures as previously described (i.e., body mass, HR, BLa concentration, and BGl concentration), participants commenced a 5 min warm up on the treadmill at 8 km h^−1^. Immediately after completing the warm up, participants then ran for ~60 min at a running velocity corresponding to 75% V^·^O_2PEAK_ (NB: two participants were unable to complete the full 60 mins on their first trial; however, they repeated the exact same duration for their second trial, hence, the mean of 58 ± 4 min vs. 58 ± 4 min reported in Section 2.2). This intensity was chosen as the energy expenditure in soccer that has been reported to correspond with 75% maximal aerobic power [28]. Every 15 min during exercise (e.g., 15, 30, 45, and 60 min), participants were given a 20 ml bolus of tap water for thermal comfort. Ratings of perceived exertion based on overall cardiovascular strain (RPE_O_; 6–20; [39]) and localised to the leg musculature (RPE_L_; 0–10; [40]) were collected every 10 min. Immediately following this, participants performed repeated 1 min intervals at a velocity corresponding to 90% V^·^O_2PEAK_, interspersed with 1 min active recovery periods at 5 km h^−1^ (walking pace), until volitional exhaustion. At the end of each 1 min interval at 90% V^·^O_2PEAK_, HR, RPE_O_, and RPE_L_ were recorded. This protocol was selected because it replicated the activity patterns and metabolic stress of speed endurance training sessions and was therefore relevant to individuals who competed in invasion sports such as soccer [41]. Upon completion of exercise, participants completed 2 min active recovery, after which body mass was remeasured and further urine, BLa, and BGl samples were collected and analysed as previously indicated.

Participants subsequently recovered for 2 h in a semisupine position. During this period, they were provided with two litres of tap water to ingest ad libitum. The mean amount consumed during familiarisation sessions (1.38 ± 0.51 L) was subsequently used to calculate the volume of fluid provided during experimental trials. Additionally, after 30 min recovery, participants were given a standardised cereal bar (Nature Value ™ Crunchy Oats and Honey Bar, General Mills, Uxbridge, UK) that contained 192 kcal (850 kJ) and constituted 7.2 g of fat (1.0 g saturated), 27.1 g carbohydrates (11.9 g sugar), 3.4 g protein, and 2.4 g of fibre. 

After the two-hour recovery period, further urine, BLa, and BGl samples were collected and analysed as previously indicated. Baseline HR was then recorded prior to commencing a 5 min warm up at 8 km h^−1^. Subsequently, participants then ran for 20 min at a running velocity corresponding 75% V^·^O_2PEAK_. For our primary performance outcome measure, the same pattern of intermittent running until volitional exhaustion (e.g., 1 min at 90% V^·^O_2PEAK_ followed by 1 min active recovery at 5 km h^−1^) was then completed a final time with HR, RPE_O_, and RPE_L_ collected at the end of each interval at 90% V^·^O_2PEAK_. Upon completion of exercise, participants completed 2 min active recovery, after which body mass was measured a final time, and further urine, BLa, and BGl samples were collected and analysed as previously indicated. Participants were then free to leave the laboratory.

### 4.6. Experimental Trials

Experimental trials were identical to familiarisation trials with one exception. During recovery, participants were provided with 1.38 ± 0.51 L of either deep ocean mineral water (DOM; PDO Biotech, Taiwan) or a taste-matched placebo (Table 6). Treatments were administered double-blind.

### 4.7. Statistical Analysis

Data were analysed using IBM SPSS (v24, IBM Corp, Armonk, NY, USA). For all data, normality (via Shapiro–Wilk’s test) and homogeneity of variance/sphericity (via Mauchly’s test) were checked prior to choosing the appropriate statistical tests. With the exception of the exercise capacity data (Wilcoxon Signed Ranks Test), a number of two-way (treatment, time) repeated measure ANOVAs were subsequently conducted. If sphericity was violated, degrees of freedom were corrected using Greenhouse-Geisser values, and Bonferroni adjusted pairwise comparisons were applied. Additionally, Tukeys’ HSD post hoc analysis was undertaken for interactions by calculating the difference required between means for a minimum level of P = 0.05 [42]. Data were analysed and quantified using effect sizes (ESs) and P values (P ≤ 0.05). For ANOVA main effects and interactions, the ES was reported as the partial η^2^ (Pη^2^). Otherwise, for normally distributed data, the ES (d) was calculated using the difference in means divided by the pooled standard deviation (SD) of the compared values [43] with 95% confidence intervals (CI) reported and adjusted for bias [44,45]. For non-normally distributed data, the ES (r) was calculated as Z/√ n [46]. Unless otherwise stated, data were presented as mean ± SD.

## Figures and Tables

**Table 1:** Group mean cardiovascular and perceptual data during initial ~60 min running at 75% V^·^O_2PEAK_.

Time	10 min		20 min		30 min		40 min		50 min ^#,+^		60 min *^,#,+^	
Treatment	DOM	PLA	DOM	PLA	DOM	PLA	DOM	PLA	DOM	PLA	DOM	PLA
HR (beats·min^−1^)	150	156	157	159	162	167	164	166	167	168	170	171
SD	10	16	9	16	8	14	9	13	7	12	8	12
RPE_L_	2.7	3.1	3.8	3.9	4.3	4.3	5.2	5.6	5.9	6.9	6.3	7.4
SD	1.0	1.3	1.2	1.4	1.2	1.2	2.0	1.6	2.0	1.9	1.9	1.9
RPE_O_	10.7	11.6	12.7	12.7	13.6	14.0	14.3	15.1	15.3	16.2	15.7	17.1
SD	2.2	1.8	1.1	1.3	1.2	0.9	1.9	1.9	2.0	2.0	2.1	2.4

DOM = deep ocean minerals; PLA = placebo; HR = heart rate; RPE_L_ = ratings of perceived exertion localised to the legs (0–10); RPE_O_ = ratings of perceived exertion related to overall cardiovascular strain (6–20). * > HR vs. 10, 20, and 40 min (P < 0.02); ^#^ > RPE_L_ vs. 10, 20, 30, and 40 min (P ≤ 0.01); and ^+^ > RPE_O_ vs. 10, 20, 30, and 40 min (P < 0.02).

**Table 2:** Group mean cardiovascular and perceptual data during 20 min running at 75% V^·^O_2PEAK_ post-recovery.

Time	5 min		10 min ^#^		15 min *		20 min *	
Treatment	DOM	PLA	DOM	PLA	DOM	PLA	DOM	PLA
HR (beats·min^−1^)	157	160	163	163	166	167	168	169
SD	10	11	12	13	11	12	10	11
RPE_L_	5.0	5.2	5.8	5.9	6.6	6.9	6.9	7.1
SD	1.5	1.2	2.2	1.4	1.9	1.5	2.2	1.5
RPE_O_	12.9	13.1	13.8	14.1	15.1	15.2	15.8	16.0
SD	1.4	1.3	1.4	1.5	1.8	1.2	2.4	2.1

HR = Heart rate; RPE_L_ = ratings of perceived exertion localised to the legs (0–10); RPE_O_ = ratings of perceived exertion related to overall cardiovascular strain (6–20). * > values after 5 and 10 min for all variables (P ≤ 0.01). ^#^ > values after 5 min for all variables (P < 0.05).

**Table 3:** Urine osmolality (mOsm/kg^−1^) over time.

Time	Baseline		Post Initial bout of Running at 75% V^·^O_2PEAK_		Post-Recovery		Post-Exercise Capacity	
Treatment	DOM	PLA	DOM	PLA	DOM	PLA	DOM	PLA
P1	590	560	900	730	910	830	900	800
P2	420	300	920	360	660	90	600	90
P3	400	570	1090	1110	210	80	200	90
P4	590	600	230	610	780	860	760	850
P5	130	600	230	770	100	290	50	280
P6	180	430	180	350	170	40	180	20
P7	580	120	510	280	320	300	300	270
P8	180	600	540	20	20	10	20	10
P9	150	540	400	580	230	200	200	180
Mean	358	480	556	534	378	300	357	288
SD	200	168	338	321	321	326	318	320

DOM = deep ocean minerals; PLA = placebo.

**Table 4:** Body mass (kg) over time.

Time	Baseline *^,#^		Post Initial bout of Running at 75% V^·^O_2PEAK_		Post-Recovery		Post-Exercise Capacity Test	
Treatment	DOM	PLA	DOM	PLA	DOM	PLA	DOM	PLA
P1	90.0	90.0	89.5	89.8	89.5	90.0	88.5	90.0
P2	76.0	76.0	75.0	75.5	75.0	75.5	75.0	75.5
P3	96.0	95.0	94.5	94.5	95.0	95.0	95.0	95.0
P4	76.0	76.0	75.0	75.5	75.5	76.0	75.5	75.5
P5	66.0	66.0	65.5	65.5	65.5	65.5	65.5	65.5
P6	74.0	72.5	73.0	72.0	73.0	72.0	73.0	72.0
P7	66.0	66.0	65.0	65.5	65.0	65.5	65.0	65.5
P8	83.0	83.0	82.5	82.5	82.5	82.5	82.5	82.5
P9	94.0	94.0	93.5	93.5	93.5	93.5	93.0	93.5
Mean	80.1	79.8	79.3	79.4	79.4	79.5	79.2	79.4
SD	11.3	11.2	11.3	11.3	11.3	11.4	11.2	11.4

DOM = deep ocean minerals; PLA = placebo; * > than all other time points, P < 0.001; and ^#^ DOM > PLA, P < 0.05.

**Table:** 

Time	Baseline		Post Initial bout of Running at 75% V^·^O_2PEAK_ *		Post-Recovery *		Post-Exercise Capacity Test *	
Treatment	DOM	PLA	DOM	PLA	DOM	PLA	DOM	PLA
P1	1.4	1.0	2.4	2.5	2.4	1.4	1.3	1.6
P2	0.9	0.9	1.2	1.7	2.3	3.1	1.6	1.7
P3	1.0	1.1	5.4	5.6	1.5	3.2	1.9	1.6
P4	1.1	1.0	5.4	3.4	3.7	1.2	3.5	2.3
P5	0.6	0.9	1.9	3.8	1.4	1.6	1.9	1.9
P6	0.9	1.2	2.3	3.5	1.7	2.1	2.0	2.9
P7	0.7	0.6	3.8	1.4	1.1	1.7	2.3	2.6
P8	1.0	1.2	7.5	3.5	2.4	2.0	4.6	3.5
P9	0.8	1.0	1.8	2.1	1.1	1.8	1.6	2.3
Mean	0.9	1.0	3.5	3.1	2.0	2.0	2.3	2.3
SD	0.2	0.2	2.1	1.3	0.8	0.7	1.1	0.7

DOM = deep ocean minerals; PLA = placebo; and * > Baseline (P < 0.01).

**Table:** 

Time	Baseline		Post Initial bout of Running at 75% V^·^O_2PEAK_		Post-Recovery		Post-Exercise Capacity Test	
Treatment	DOM	PLA	DOM	PLA	DOM	PLA	DOM	PLA
P1	4.4	3.6	5.6	4.3	4.8	4.8	5.3	4.3
P2	13.1	11.7	5.7	7.0	9.4	15.3	4.1	9.0
P3	4.6	4.6	4.3	4.2	5.5	5.7	3.9	3.8
P4	4.8	5.0	4.6	4.8	3.2	3.7	3.2	4.9
P5	3.7	4.2	2.9	3.5	4.3	6.2	3.6	2.9
P6	3.7	5.0	4.1	5.0	3.8	4.0	4.1	3.7
P7	3.3	3.1	3.1	2.9	6.0	4.6	2.5	2.4
P8	4.7	5.1	4.7	4.2	5.8	4.8	4.8	4.4
P9	4.2	4.6	3.4	4.6	3.5	5.4	3.4	5.4
Mean	5.1	5.2	4.3	4.5	5.1	6.0	3.9	4.5
SD	3.0	2.5	1.0	1.2	1.9	3.5	0.9	1.9

DOM = deep ocean minerals; PLA = placebo.

**Table:** 

Ingredients	Composition (%)	
	DOM	PLA
Purified Water (RO)	93.25	93.65
Sucrose	6.00	6.00
Deep Ocean Mineral (DOM) Concentrate	0.40	N/A
Flavour (Grapefruit, Citrus Paradise)	0.12	0.12
Citric Acid	0.12	0.12
Concentrated Lemon Juice	0.05	0.05
Calcium Lactate	0.04	0.04
Potassium Chloride	0.02	0.02
Sodium Chloride	0.01	0.01

Key: DOM = deep ocean mineral water; PLA = placebo.

**Table:** 

Element	mg/L (Undiluted)	mg/L (Diluted)
Magnesium (Mg)	42,797	170.70
Sodium (Na)	13,700	54.64
Calcium (Ca)	56	0.22
Potassium (K)	14,200	56.64
Boron (B)	135	0.54
Rubidium (Rb)	4.7	0.02

Note: Diluted = Undiluted × 0.4%; Overall hardness of DOM = 704.
